# Advances in Understanding the Relationship Among Brain, Physical Function, and Exercise Performance

**DOI:** 10.3390/brainsci16070686

**Published:** 2026-06-29

**Authors:** Luca Petrigna

**Affiliations:** Department of Biomedical and Biotechnological Sciences, Section of Anatomy, Histology and Movement Science, School of Medicine, University of Catania, Via S. Sofia n◦97, 95123 Catania, Italy; luca.petrigna@unict.it

## 1. Introduction

The research on the relationship among brain, physical function, and exercise performance is developing faster than previously, with an increasing number of articles year after year. This topic is old but relevant. Giovenale, between the years 50 and 140 A.D., wrote the Latin phrase “mens sana in corpore sano”, which means “healthy mind in a healthy body” [1]. One of the first scientific studies on this topic was published in 1977 [2], and, in the years since, the number of scientific articles on this topic has constantly increased. Nowadays, the scientific literature states that regular physical activity and exercise is associated with better cognitive function [3] in children [4], adolescents [5], adults, older adults, and people with cognitive degeneration [6]. Similarly, sport activities have a positive correlation with cognitive function [7,8]. It has emerged how all forms of movement are fundamental at all ages and in all conditions.

Exercise during development improves cognitive performance and abilities [4,9]. Memory and executive function are positively influenced in this population [10], as are academic achievements [4]. Positive outcomes are also observed in children with attention deficit hyperactivity disorder [11] and with neurodevelopmental disorders [12] or autism spectrum disorder [13]. Similar effects occur in adults [14] and healthy older adults [15]. In people with neurodegenerative pathologies, physical exercise seems to have a protective role [16,17] and a positive effect on cognitive function [18,19]. Physical activity has a positive effect on the circulating levels of neurotrophic factors, brain-derived neurotrophic factor and neurotrophin-4 [20]. Physical activity reduces the circulating levels of oxidative stress markers in nervous system injuries [21]. Furthermore, physical activity seems to act through neuroplasticity [22].

It is important to highlight how the positive interaction between cognitive function and movement has been investigated in the short and long term. Both the acute effects [23] and the chronic adaptations [24] have been continuously presented in the literature, and the research is still moving forward, reducing the gap between the scientific context and the everyday world. To obtain positive results, it is fundamental to choose the most suitable dose of movement (in terms of frequency, intensity, time, type, progression, and volume), and, in this regard, the American College of Sports Medicine (ACSM) guidelines [25] can help us and to determine the ideal cognitive load [26]. An important aspect that should be considered is the environment. Indeed, in the last years, the literature has been growing on the importance of practicing activities in a natural setting [27], meaning it is better if these activities are outdoors [28]. Another important aspect to consider is related to new technologies and social media, which are negatively impacting the mental health of the population [29]. Future studies are still required to better understand these topics.

This Special Issue aims to provide an overview of the topic, to present new approaches and discuss old ones, considering different populations in terms of age and condition. Different methodologies were adopted, but the main aim of the published articles was prioritizing the physiological and cognitive wellness of the population. Ten articles have been included: six original articles and four reviews are online. All the articles evaluated the effects of physical activity and exercise on cognitive function, directly and indirectly, in different populations, both healthy and with pathologies.

## 2. An Overview of the Published Articles

In this Special Issue, four articles focus on people with pathologies or impairments such as mild cognitive impairment, Parkinson’s disease, history of concussion, and intellectual disabilities. Different intervention methodologies were investigated, such as high-velocity and low-resistance (high-cadence) interval cycling, aerobic exercise, and virtual reality with dancing. The outcomes investigated were cognitive function, executive and motor performance, and risk of falls. Three originals studies and one systematic review and meta-analysis of randomized controlled trials were conducted. Bardopoulou and colleagues focused on people with mild cognitive impairment. The authors investigated how cycling in continuous aerobic/moderate-intensity and high-velocity/low-resistance (high-cadence) intervals impacted cognitive parameters and executive performance. The authors found that, in this population, the high-velocity, low-resistance (high-cadence) intervals cycling was more impactful than continuous aerobic exercise. Delgado-Anguiano and colleagues studied people with idiopathic Parkinson’s disease at different stages. The authors effectively adopted virtual reality with dancing as a tool to improve walking speed, postural stability, and reduce the risk of falls. Young and Staines examined people with a history of concussion. In this case, acute aerobic exercise at moderate intensity was adopted, and the authors found positive effects on accuracy. The last study was a systematic review and meta-analysis on people with special needs and intellectual disabilities. The aim of this review is important: Zhang and colleagues tried to identify the optimal exercise prescription for this population. The authors stated that exercise significantly improved cognitive function in people with intellectual disabilities. Different detailed information regarding the exercise prescription is provided by Zhang and colleagues. Indications are also presented according to age.

Two original articles focus on athletic people: one on gym users and sportive people, the second one on healthy endurance athletes. The variables investigated were resting-state electroencephalography and neuromuscular performance. Fan and colleagues evaluated how motivational music impacts physical performance. An exhaustion test with and without music was conducted, and the authors discovered that music prolongs the time to exhaustion. A thorough description of the brain mechanisms behind this finding are well-presented within the article. The second study by Petrigna and colleagues is exploratory. The authors studied if differences exist between people who perform gym activities and sportive open-skills individuals in terms of neuromuscular performance. No statistically significant differences were detected. The study provides useful feedback for future investigations on this topic.

The last four studies considered healthy people, children, young adults, adults, and older adults, focusing on response inhibition, working memory, cognitive flexibility and function, and motor performance. The tools adopted were electroencephalography and dual tasking. These four studies include one original study and three reviews: one narrative, one scoping, and one systematic with a meta-analysis. Sugawara and colleagues examined the Go/NoGo task and its effects. The authors found that even though the repetition of this task did not enhance behavioral performance, a reduction in neural activity was noted, suggesting some neurophysiological adaptations. Guzmán-Muñoz and colleagues, through a narrative review, stated that the practice of acute physical activity has a positive effect on improving inhibitory control, working memory, and cognitive flexibility. Chronic physical activity leads to structural and functional adaptations. A detailed description of the adaptation is provided within the manuscript. Dual-task training is examined in the scoping review by Petrigna and colleagues. In the study, the authors proposed a standard operating procedure as a training intervention in cognitive dual-tasking, suggesting two 60 min sessions per week for at least 12 weeks. The last study included in this Special Issue is by Liu and colleagues, which demonstrate that the flipped classroom model is better than traditional teaching in improving cognitive and motor performance. A detailed explanation and overview of the topic are presented in the systematic review and meta-analysis.

## 3. Conclusions

The findings in this Special Issue show that physical and cognitive health are equally important and strongly interconnected. The benefits at the physical level are transferable to the mental level and vice versa. The studies included, even if on different populations (in terms of health status, age, or physical background), with different interventions and outcomes, aligned in that people must train both their body and their brain.

The topic is still new; it can be further developed. Future studies could deeply investigate how new technologies impact the lives of people from physical and cognitive points of view and how these technologies can be useful in improving the quality of life of the individual. Studies could better investigate brain functioning under different types of training or cognitive conditions. Studies could focus their attention on children during development. This Special Issue will provide some useful data and feedback for further studies on the topic.
